# Conformational dynamics control assembly of an extremely long bacteriophage tail tube

**DOI:** 10.1016/j.jbc.2023.103021

**Published:** 2023-02-13

**Authors:** Emily Agnello, Joshua Pajak, Xingchen Liu, Brian A. Kelch

**Affiliations:** Department of Biochemistry and Molecular Biotechnology, University of Massachusetts Chan Medical School, Worcester Massachusetts, USA

**Keywords:** bacteriophage, cryo-EM, thermophile, virus assembly, molecular dynamics

## Abstract

Tail tube assembly is an essential step in the lifecycle of long-tailed bacteriophages. Limited structural and biophysical information has impeded an understanding of assembly and stability of their long, flexible tail tubes. The hyperthermophilic phage P74-26 is particularly intriguing as it has the longest tail of any known virus (nearly 1 μm) and is the most thermostable known phage. Here, we use structures of the P74-26 tail tube along with an *in vitro* system for studying tube assembly kinetics to propose the first molecular model for the tail tube assembly of long-tailed phages. Our high-resolution cryo-EM structure provides insight into how the P74-26 phage assembles through flexible loops that fit into neighboring rings through tight “ball-and-socket”-like interactions. Guided by this structure, and in combination with mutational, light scattering, and molecular dynamics simulations data, we propose a model for the assembly of conserved tube-like structures across phage and other entities possessing tail tube–like proteins. We propose that formation of a full ring promotes the adoption of a tube elongation-competent conformation among the flexible loops and their corresponding sockets, which is further stabilized by an adjacent ring. Tail assembly is controlled by the cooperative interaction of dynamic intraring and interring contacts. Given the structural conservation among tail tube proteins and tail-like structures, our model can explain the mechanism of high-fidelity assembly of long, stable tubes.

Bacteriophages are ubiquitous viruses that selectively and specifically infect their bacterial host. The overwhelming majority of phages are of the order *Caudovirales*, which consist of an icosahedral capsid that contains the double-stranded DNA genome and a tail. The tail is essential for host recognition and viral attachment and therefore successful infections, because it serves as the conduit through which the genome travels from the capsid to the host. Tails have morphologically distinct features that further subclassify them into three families: (1) the short-tailed *Podoviridae*, (2) the long, contractile-tailed Myoviridae, and (3) the long, noncontractile-tailed Siphoviridae. The tail tubes of long-tailed phage (∼85% of all phage) share a common architecture and conserved constituent proteins, suggesting similar principles underlie the assembly and stability of both classes of tails. These tails minimally consist of a tape measure protein (TMP), a complex of tail tip proteins thought to initiate tail assembly, and the tail tube protein (TTP) that polymerizes to form the majority of the tube architecture (1, 2, 3, 4). There is a clear shared homology and evolutionary origin between TTPs of long-tailed phage, proteins of the bacterial type VI secretion system, and bacteriocins (5, 6).

Owing to their long, flexible nature, structural information on tail tubes of siphophages has primarily been limited to pseudo-atomic models using monomeric crystal or NMR structures fit into low-resolution cryo-EM density (7, 8, 9, 10, 11, 12). More recently, however, cryo-EM studies have begun to elucidate how TTP is organized in tail tubes (13, 14, 15), revealing a conserved fold for TTP, with helically stacked hexameric rings creating a tube whose lumen is occupied by TMP. However, the lack of high-resolution structural information of assembled tubes, combined with a lack of studies revealing assembly kinetics, has limited our understanding of tail tube assembly. To understand the assembly and stability of tail-like structures of phage and other phage-related entities, critical questions regarding conformational changes, biochemical interactions, assembly kinetics, and assembly fidelity remain.

While most siphovirus tails range in length from about 50 to 200 nm (5), a hyperthermophilic phage called P74-26 stands out with the longest tail of any known virus at almost a micron in length (Fig. 1*A*). P74-26 infects the gram-negative bacterium *Thermus thermophilus*, which grows at an optimal temperature of 65 °C (16, 17). Owing to the extreme conditions that this phage must endure, it has been characterized as the most stable Caudovirus known (18). Here, we report a 2.7 Å structure of the P74-26 tail tube using cryo-EM. We find that the P74-26 TTP forms rings that are trimeric rather than hexameric and assembles using an abundance of hydrophobic and electrostatic interactions. Purified P74-26 spontaneously forms flexible tubes that are nearly structurally identical to tails of intact phage. Equipped with the ability to reconstitute tube assembly *in vitro*, we probed protomer and ring interactions through kinetic experiments, mutational analysis, and molecular dynamics (MD) simulations to propose a mechanism for tail tube assembly. We find that assembly is governed by the formation of ball-and-socket joints, which results in cooperative formation of intraring and interring interactions that overcome autoinhibitory barriers in the monomeric TTP. We propose a model for the formation and growth of tail tubes that can explain the high-fidelity mechanism for the assembly of long, stable tubes.

**Figure 1:**
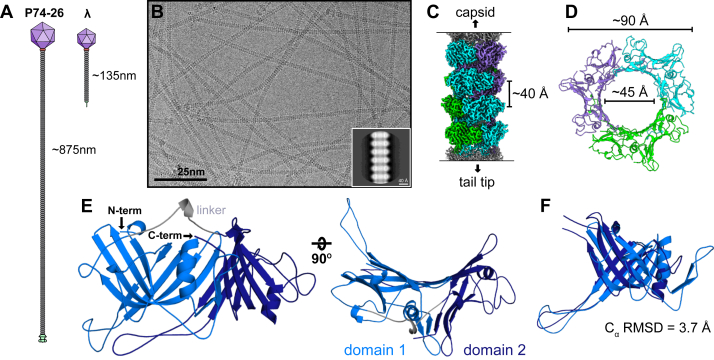
**P74**-**26 tail tube structure.***A*, schematic of siphoviruses P74-26 (*left*) and λ (*right*) illustrating the stark difference in tail length. *B*, representative cryo-EM micrograph of P74-26 virions, with an example 2D class (inset). *C*, cryo-EM reconstruction of a segment of tail tube, with four stacked rings highlighted. *D*, a single trimeric ring viewed top-down. *E*, TTP domain architecture. Each subunit consists of two structurally similar domains. Domain 1 (*light blue*) is connected to domain 2 (*dark blue*) by a linker (*gray*). *F*, overlay of the two domains showing their structural similarity. TTP, tail tube protein.

## Results

### Structure of the P74-26 virion tail tube

#### Overall architecture

We determined the structure of the P74-26 tail tube using cryo-EM (PDB ID 8ED0). Micrographs of purified P74-26 virions revealed flexible tails with a stacked ring pattern (Fig. 1*B*). Because of the flexible nature of the tails, traditional single-particle analysis could not be used; instead, we picked sections of tail with ∼6 rings per “particle” and used a segmented helical reconstruction approach with the helical symmetry tools in CryoSPARC (19) (Fig. S1). Iterative symmetry searches and helical refinements revealed that the P74-26 tail tube has an overall C3 symmetry, with three protomers per ring. C3 symmetry has been seen twice before in the tail tubes of T5 and ΦCbK phage (9, 20, 21). The tube is helical, with a rise of 40.25 Å and a twist of −44.75° (Figs. 1*C* and S1). The left-handed helical twist is rare for long-tailed phages, as the majority of tail tubes are right-handed (22, 23). Our final reconstruction has a global resolution of 2.72-Å, according to the gold standard 0.143 Fourier Shell Correlation criteria (Fig. S2, *A* and *C*). This resolution allowed us to clearly observe every residue in the TTP from the N-terminus to the C-terminus.

Using this reconstruction, we built an atomic model of the gp93 TTP *de novo* in its entirety, which we were able to trace unambiguously. We then fit twelve copies of the TTP atomic model into the density of the central four of the six rings in the reconstruction (the two remaining rings at the periphery of the map are of much lower resolution). The diameters of the outer and inner surface of the tube are ∼90 Å and ∼45 Å, respectively (Fig. 1*D*), consistent with other known tail tubes. In some phages, the tail tube has exterior protrusions, such as the immunoglobulin-like (Ig-like) domains seen in T5, λ, YSD1, and Araucaria (9, 10, 13, 24). The P74-26 tail tube, however, has a markedly smooth outer surface with no additional domains or protrusions (Fig. 1*C*). There is density that runs through the center of the tube, which presumably corresponds to the tail’s TMP, gp95 (Fig. S2*D*). The local resolution for this density in the lumen is much lower than the rest of the map, likely due to the segmented method used for reconstruction. We attempted to further resolve the secondary structure in the TMP density but were unsuccessful and left this density unmodeled. A closer look at the surface of the tail tube lumen reveals surprisingly net neutral electrostatics (see Supporting Text and Fig. S3)

**Figure 2:**
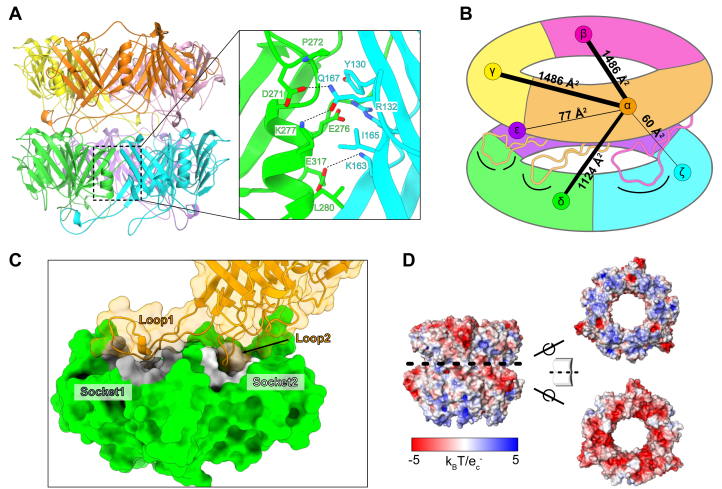
**Intraring and inter**r**ing interactions.***A*, the primary intraring interface between two subunits (*green and cyan*) within a ring. *B*, schematic quantifying interactions from a single subunit (α) to illustrate the extensive, cooperative network. Numbers and line widths (not lengths) correspond to quantification of the interactions between α and neighboring subunits as calculated by the PDBePISA server (Tables S3 and S4). *C*, surface representation of two subunits reveals a ball and socket geometry between rings. A single subunit (*orange*) has two loops (Loop1 and Loop2) that fit into sockets (Socket1 - *gray* and Socket2 - *white*) of a subunit in the ring below it (*green*). *D*, surface electrostatics of ring interfaces demonstrate an important role for electrostatics in interring interactions.

Each P74-26 TTP subunit consists of two β-sandwich domains: an N-terminal domain (domain 1) and a C-terminal domain (domain 2), connected by a linker. Each domain has the same β-sandwich fold as other TTPs, indicating that it is evolutionarily related to other TTPs despite the difference in overall symmetry and very low sequence similarity (∼10% identity). Much like single-domain TTPs, each β-sandwich domain contains a long hairpin that emanates from the “bottom” of the TTP subunit; we term these loops as “Loop1” (from domain 1) and “Loop2” (from domain 2) (Fig. 2*C*). The two domains are structurally similar to each other (C_ɑ_ RMSD of ∼3.7 Å over 113 out of 174 residues; Fig. 1*F*), despite having very little sequence similarity (16% identity). Therefore, the two-domain architecture of P74-26 TTP likely arose through an ancient gene duplication and fusion event. Thus, despite the C3 symmetry, the overall ring can be considered pseudohexameric, explaining the similarity to hexameric tail tubes of most long-tailed phages. Furthermore, the N-terminal domain of one subunit is related to the C-terminal domain of a subunit in an adjacent ring in a right-handed fashion with a twist of ∼19°, consistent with the helical twist of right-handed hexameric tail tubes. Similar pseudosymmetry is also seen in siphophage T5, where each subunit similarly consists of two β-sandwich domains (Fig. S4) (9). As in T5, each P74-26 TTP domain is structurally similar to the TTP of other phage and R-pyocins, illustrating a shared evolutionary lineage (5, 6, 9).

#### Interring and intraring interactions

The tail tube is held together by two types of interfaces: within a ring (intraring) and between rings (interring). Within each ring, subunits are arranged head-to-tail into a trimeric ring, reminiscent of DNA polymerase sliding clamps (ring-shaped proteins that also evolved through domain duplications) (25, 26, 27). Each subunit contacts six subunits interring (three “below” and three “above”.) The intraring interaction area is slightly larger than the interring (3 × 1486 Å^2^ = 4458 Å^2^ *versus* 3 × 1261 Å^2^ = 3783 Å^2^; Fig. 2*B* and Table S4).

The intraring interactions come from two interfaces: a direct interaction between the two β-sandwich domains of adjacent subunits and another interface between Loop1 of one subunit and the underside of the adjacent subunit’s domain 2 (Fig. 2*A*). Both interfaces are particularly rich in hydrophobic interactions. For example, Met1, Tyr130, and Phe123 in domain 1 bind to Pro272, Ala253, Ala 273, Val322, and Ile328 of domain 2, while Met55 in Loop1 inserts into a hydrophobic pocket formed by Leu108, Val244, Ile308, and Ala336 of domain 2. We further note that the N-terminal methionine directly contributes to intraring interactions, providing a structural explanation for previous work showing that the flexible N-terminus controls tail assembly in other phages (8, 10).

Nearly all interring interactions are mediated by Loop1 and Loop2. Loop1 makes slightly more extensive interring contacts than Loop2 (811.9 Å^2^ *versus* 521.6 Å^2^) (Table S4). Thus, Loop1 plays a critical role in stabilizing interactions both within rings and between rings. Each TTP subunit contacts both other subunits within the same ring as well as all subunits in both adjacent rings, creating a cooperative unit (Fig. 2*B*). The interring interactions are mediated by a ball-and-socket–like geometry, wherein the tips of Loop1 and Loop2 each fit into a socket of a subunit in the adjacent ring; Socket1 and Socket2, respectively (Fig. 2*C*). Furthermore, this interface is supported by extensive electrostatic interactions, with the surface of the loop-side harboring a net positive charge while the socket-side is negative (Fig. 2*D*). While the overall loop and socket geometry appears to be conserved across tail tubes, the P74-26 tail tube uses exaggerated ionic interactions for enhanced thermostability (see Supporting Text and Table S3).

### Tail tube protein polymerizes *in vitro*

To investigate P74-26 TTP polymerization *in vitro*, we recombinantly expressed and purified the P74-26 TTP in *Escherichia coli* (Fig. S5*A*). When we examined purified, soluble TTP by negative stain EM, we observed long structures resembling tail tubes (Fig. S5*B*). These tubes form a range of lengths but are on average much longer than virion tails, presumably because the TMP that regulates tube length during virion formation is absent (28). In addition, the *in vitro*–assembled tubes exhibit greater flexibility than virion tails (Fig. S5*D*). Thus, we posit that the TMP contributes to the stiffness of virion tails. The tube formation appears to be irreversible under all conditions tested, including a range of pH and temperatures.

To evaluate the similarity between the *in vitro*–assembled tubes and tails from intact virions, we determined the structure of the reconstituted tubes using cryo-EM (PDB ID 8EDX) to a global resolution of 2.8 Å (Figs. S2, *B* and *E* and S5*C*), using a similar helical reconstruction protocol as we implemented for virion tails. We found that *in vitro*–assembled tubes are structurally identical to the virion tail structure (C_α_ RMSD ∼ 0.11 Å across all 348 residues), except there is no density for TMP running through the center of the tube, as expected (Fig. S2*E*). Therefore, the *in vitro* assembly of TTP into tubes establishes this system as a useful tool for revealing the mechanism of tail tube polymerization.

**Figure 3:**
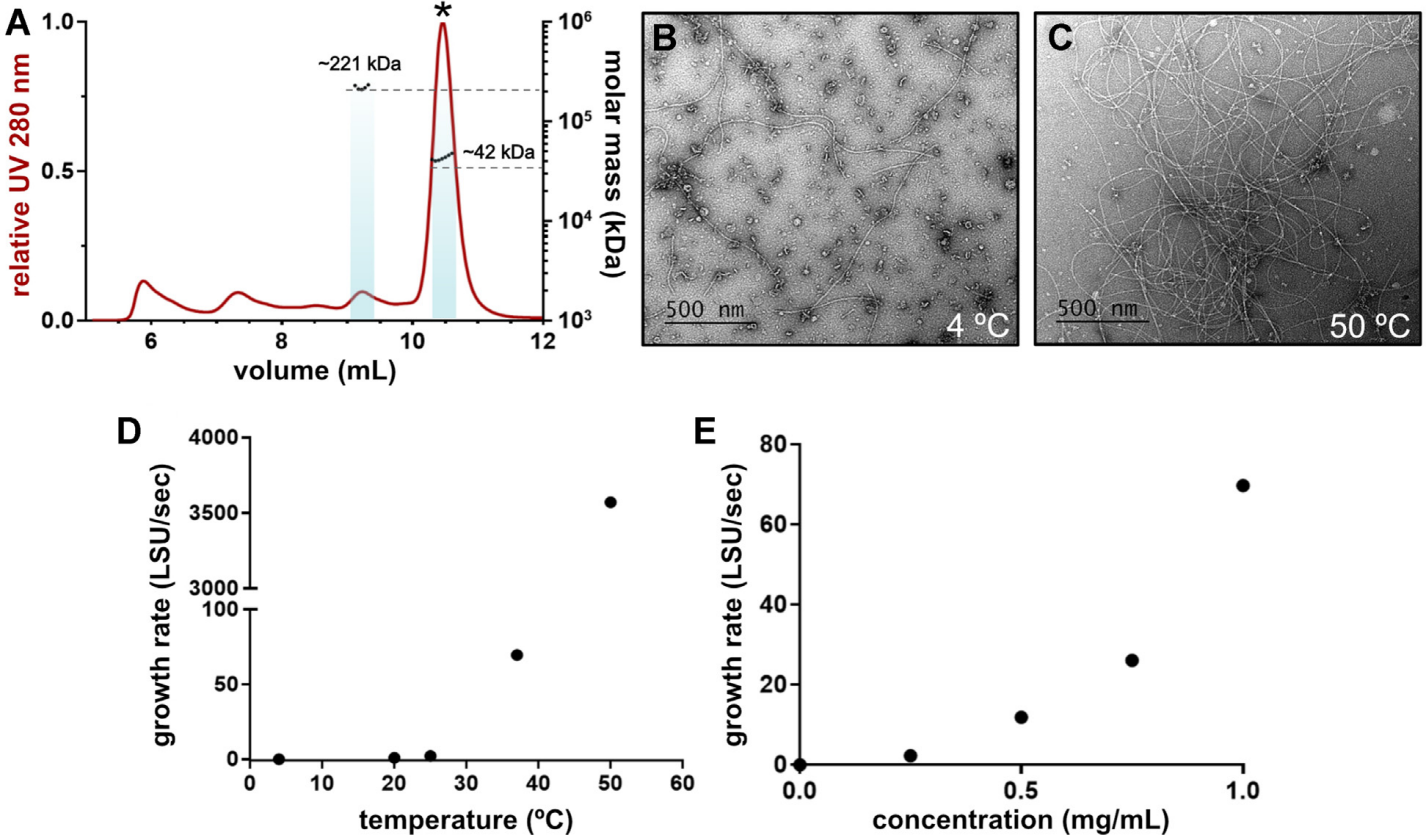
**Properties of *in vitro* TTP assembly.***A*, SEC-MALS of filtered TTP, displaying the UV trace (*red*) and calculated molecular weights (*black dots*) with an average calculated molecular weight of 42.3 ± 0.347 kDa and 221 ± 8.18 kDa 3.7%. *Dashed lines* display the true molecular weights for a monomer (∼37.7 kDa) and a hexamer (∼226 kDa). *B* and *C*, negative stain images of *in vitr*o–assembled tubes incubated for 3 h at 4 °C (*B*) or 50 °C (*C*) indicate tube formation is enhanced by heat. *D*, calculations of growth rates in Light Scattering Units per second, detailed in Figs. S6 and S7. Tube formation is nonlinearly accelerated by higher temperature. *E*, tube growth rates increase nonlinearly by higher TTP concentration at 37 °C. SEC-MALS, size-exclusion chromatography with multi-angle light scattering; TTP, tail tube protein.

To understand how TTP polymerization occurs, we asked what the dominant oligomerization state of TTP in solution is. We filtered purified TTP to remove any spontaneously polymerized tubes and analyzed the flow-through using size exclusion chromatography with multi-angle light scattering (SEC-MALS), which measures absolute molecular mass of particles in solution. We observe that the majority of soluble TTP is monomeric in solution (∼76% mass fraction; Fig. 3*A*). This observation is not unexpected, as TTP from other phages are primarily monomeric in solution as well (9, 13, 29). We also observe a minor peak whose molecular weight is consistent with a hexamer of TTP (mass fraction ∼6%).

Because of the thermophilic nature of P74-26, we explored the temperature dependence of our *in vitro*–assembled tube formation. We applied purified TTP to negative stain grids after incubation at 4 °C and 50 °C for 3 h. There was an increase in the number of tubes per image as the temperature increased, suggesting tube assembly was promoted by higher temperatures (Fig. 3, *B* and *C*).

As sample application in negative stain EM can be variable, we sought to establish a more quantifiable method for measuring tube assembly under a range of conditions. To do so, we took advantage of the mass increase that accompanies tube growth, increasing light scattering as assembly progresses. Using a light scattering assay, we monitored tube assembly from purified TTP at 4 °C, 20 °C, 25 °C, 37 °C, and 50 °C. Consistent with the negative stain results, tube formation increased with temperature (Fig. S6*A*). Kinetically, the assembly of tail tubes over time at 4 °C and 20 °C progressed linearly, while the 37 °C displayed a sigmoidal (S-shaped) shape and the 50 °C condition exhibited a fast initial burst of assembly followed by a plateau (Fig. S6*A*). To quantitatively compare rates of tube formation across this temperature range, we calculated the rates during the fast-phase of tube growth for each condition (Fig. 3*D*). While assembly of TTP tubes occurred slowly over 3 h at 20 °C, assembly occurred much faster at 37 °C and even faster still at 50 °C, with rates ∼68× and 3,516× the 20 °C rate, respectively (Fig. S6*C*). The relationship between temperature and growth rate is nonlinear and demonstrates a steep temperature dependence.

Using this same assay, we then measured the concentration dependence of tube formation at 37 °C. The curves of light scattering *versus* time were sigmoidal in shape (Fig. S7*A*), so we again focused on the fast rate of the curves confirming that increased concentrations result in a faster rate of assembly (Fig. S7*D*). We then plotted the rates of assembly against TTP concentration and found that the concentration dependence is nonlinear (Fig. 3*E*). This indicates a complex relationship with TTP concentration and suggests that the initial seeding and tube elongation may proceed with different kinetics.

### Mutational analysis of tube formation

**Figure 4:**
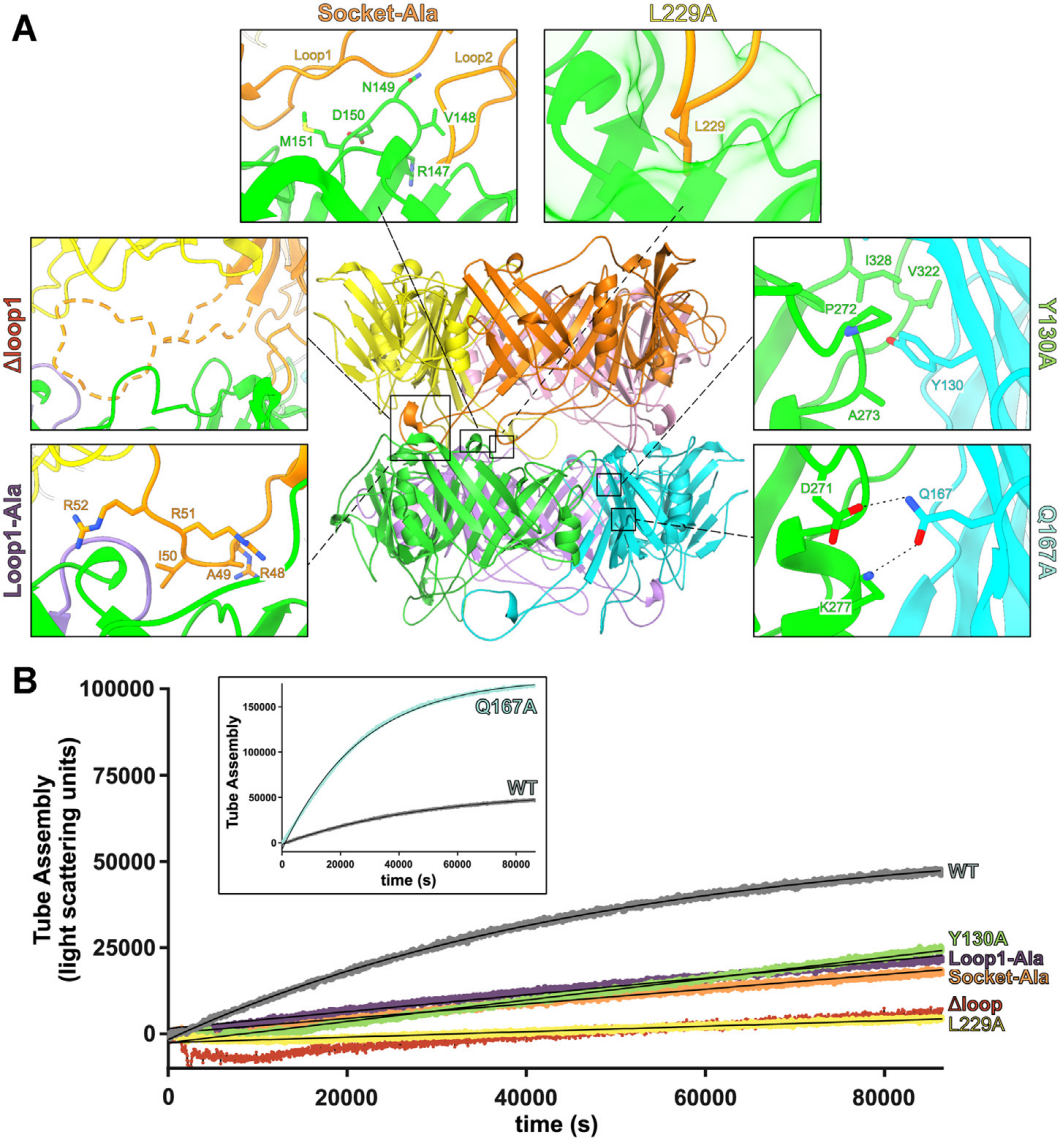
***In vitro* mutational analysis of tube assembly.***A*, location of the chosen TTP mutations in the context of the tube (*cente*r) and zoomed-in views of residues selected for mutation. *B*, light scattering curves of TTP variant tube formation at 1 mg/ml over time, with higher light scattering intensity values (counts per second) indicating a more highly assembled state. Variant Q167A (inset, on a larger scale appropriate to the values) has a much quicker rate of assembly and a higher plateau, suggesting a negative regulatory role for residue Q167 in tube formation. Negative stain EM confirming assembly and a summary of rates can be found in Fig. S9. TTP, tail tube protein.

Using our cryo-EM structures, we hypothesized that the intraring and interring interactions control tube assembly. We tested whether these interactions are important for tube growth using site-directed mutagenesis and *in vitro* tube growth assays. To target intraring interactions, we generated two separate variants: the Y130A mutation (Y130 sits in a hydrophobic pocket at the interface between two subunits) and the Q167A mutation (Q167 hydrogen bonds to the adjacent subunit) (Fig. 4). To target interring interactions, we generated four separate variants (Fig. 4). The L229A mutation tests the role of L229, which sits at the tip of Loop2 and interacts with the corresponding socket on the subunit of the next ring through hydrophobic interactions. Because there are not any residues in domain 1 that make as extensive contacts as L229, we made quadruple alanine mutations at the tip of Loop1 (48-RAIRR-52 to 48-AAAAA-52; termed the Loop1-Ala variant). We also sought to disrupt the sockets, by mutating the loop that comprises the wall between the two sockets from 147-RVNDM-151 to 147-AAAAA-151 (termed the socket-Ala variant). Finally, to test the role of Loop1 and if assembly could occur with only one loop per subunit, we deleted the entire Loop1 (Q43-T65; the Δloop1 variant).

TTP variants were purified and confirmed to be primarily monomeric in solution after filtering (Fig. S8). After filtering, we monitored tube formation by light scattering. We note that these experiments were done at 20 °C rather than at 37 °C to minimize any complications from potential thermostability defects. The Δloop1 variant had drastic effects, with light scattering signal just barely above zero. Negative stain EM confirmed almost no tubes, but with the very occasional presence of short tubes, suggesting the variant is folded and capable of assembly but that assembly is severely disfavored (Fig. S9*G*). This suggests that Loop1 is required for tube assembly. The Loop1-Ala variant, however, had tube formation similar to WT, based on both light scattering and negative stain, suggesting that the necessary interactions within this loop are not specific to these interring residues (Figs. 4*B* and S9*D*). In contrast, Loop2 relies heavily on a single interaction: the L229A residue at the tip of the loop which sits in a hydrophobic pocket in Socket2. The L229A variant severely limited the rate and extent of tube formation, with a tube formation rate similar to that of the Δloop1 variant (∼10% of the WT) but with tubes still sparsely distributed in negative stain EM (Figs. 4*B* and S9*F*). Similarly, the socket-Ala variant also limited tube assembly with far less tube formation than WT (∼18% of the WT rate) but not completely abrogated (Fig. S9*E*). The intraring mutations had varying results with the Y130 residue appearing to be important to tube formation, while the Q167A variant had increased levels of tube formation, suggesting the Q167 residue may play an inhibitory or regulatory role in assembly (Figs. 4 and S9*B*). We note that the SEC-MALS data for the Q167A variant was somewhat noisy due to the increased polymerization making it difficult to isolate nonpolymerized intermediates. The data does, however, show that unpolymerized Q167A TTP is majorly trimeric as opposed to monomeric (Fig. S8). With the Q167 residue at the interface between subunits within a ring, it is clear that mutating the glutamine residue is allowing for trimer formation to occur more readily. This could be a potential explanation for the observed increase in polymerization rate, because this intermediate is more accessible in the Q167A construct than other variants. Variant assembly rates are summarized in Fig. S9*H*. This mutational analysis reveals that while intraring interactions are important, tube growth is critically dependent on interring interactions.

### Dynamics of TTP oligomers

We hypothesize that the conformational dynamics of the monomeric and/or partially assembled states primarily control the rate of assembly. This hypothesis is based on the fact that the configurational entropy of the assembled state is clearly lower than in the unassembled state. Therefore, the temperature-dependent entropic effects on rate of assembly must be primarily dictated by the unassembled state. Therefore, we used MD simulations to model the conformational dynamics of these unassembled states.

**Figure 5:**
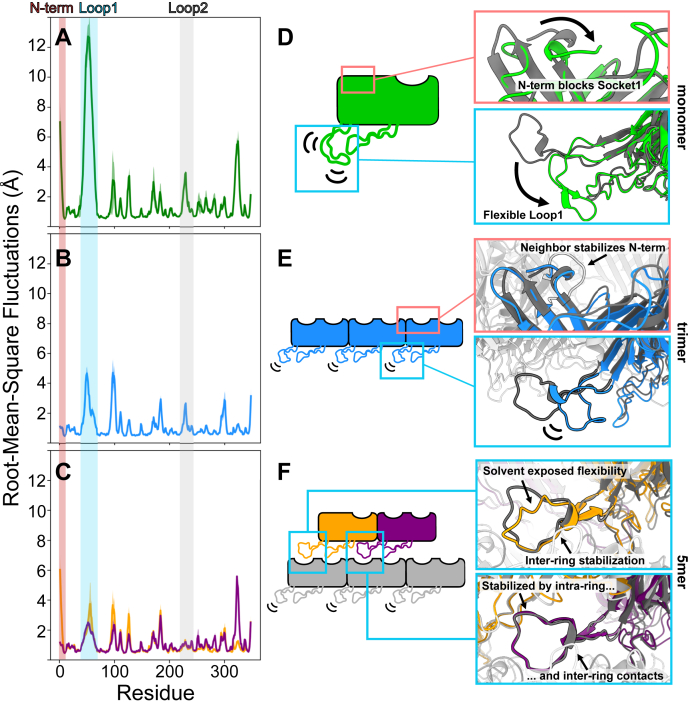
**Progressive stabilization predicted by molecular dynamic simulations.***A*–*C*, RMSF plots of monomer (*A*), trimer (*B*), or 5mer (*C*) simulations. Mean values across all replicates are lines colored according to the schematic figures shown in *D–F*, and the standard error in the mean is colored in a lighter shade. Shaded regions correspond to the N term (*red*), Loop1 (*cyan*), and Loop2 (*gray*) to highlight the stabilization of these regions at different stages of assembly. *D*–*F*, cartoon schematics of predicted dynamics with insets visualizing simulated structures (color) superimposed onto the cryo-EM reconstruction (*grayscale*). The simulated structures are representative centroids of the largest cluster of a *k = 10* means clustering of an individual simulation. These structures are provided as PDB files in the supplemental information. Subunit colors are different from previous figures in order to clarify the focus and contrast of each panel. RMSF, RMS fluctuations.

To yield insight into the preferred conformations of monomeric TTP, we performed four independent ∼2 μs MD simulations using a single subunit from our tail structure as the starting model. During the simulations, the β-sandwich domains remain relatively stable. However, Loop1 and the N-terminal residues exhibit significant flexibility and conformational changes (Fig. 5, *A* and *D*). Loop1 is highly flexible, permitting it to fold back and interact with Loop2 and the β-sandwich domain, where it is stabilized (Figs. 5*D* and S10). Further, this flexibility allows it to form secondary structural motifs (Movie S1). Thus, the conformational ensemble of Loop1 in soluble monomers is largely incompatible with the intraring- or interring-binding modes seen in the assembled tube. Moreover, Socket1 can become blocked by the N-terminal methionine, which flips ∼180° to interact with Phe33. This steric block is further stabilized by Arg2 interacting with an acidic patch consisting of Asp184, Glu185, and Glu186. Thus, we predict that the monomer state inhibits assembly due to the flexibility of Loop1 and the closing of Socket1.

To see how these autoinhibitory interactions are altered upon ring formation, we simulated a trimer ring. These simulations reveal that intraring interactions poise the trimer for tube elongation by alleviating some of the inhibitory conformations that prevent interring assembly in the monomeric state. In the trimeric state, the Met1 side chain forms part of the hydrophobic interface between subunits, locking the N-terminus into the conformation seen in tubes, with no significant blocking of Socket1 (Fig. 5, *B* and *E*). Therefore, the intraring interactions “lock” Socket1 into an open conformation that is competent for binding of future interring interactions. Similarly, Loop1 is also partially stabilized by intraring contacts (Fig. 5*B*). However, the loop remains somewhat flexible. This flexibility ultimately results in a loss of native contacts during the simulations between Loop1 and the neighboring subunit (Figs. 5*E* and S11, and Movie S2). The linchpin for Loop1 seems to be hydrophobic interactions between the top of the loop and a hydrophobic pocket of the neighboring intraring subunit; when this interaction is lost, Loop1 becomes much more flexible (Movie S2). Therefore, the formation of a trimeric ring positions Socket1 into a competent conformation, but Loop1 remains too flexible to support interring contacts.

To investigate the role of both intraring and interring contacts in potential assembly intermediates, we simulated a pentameric arrangement: a trimeric ring with two subunits of the ring above. This subunit arrangement allows us to examine how Loop1 responds to different environments, as we have a single Loop1 that is making only interring contacts, one Loop1 that is making both intraring and interring contacts, and three Loop1s that only make intraring contacts. In our pentamer simulations, Loop1 is considerably more rigid when it is stabilized by *both* intraring and interring interactions (Fig. 5*C*). Interestingly, Loop1 appears to be more stabilized by interring interactions alone than intraring interactions alone (Fig. 5, *B* and *C*). An important distinction is that the flexibility of the loop making only interring interactions is primarily in the solvent-exposed region (Fig. 5*F*), and this loop maintains native contacts better than loops stabilized by only intraring contacts (Fig. S11). Together, these simulations indicate that the vertical stacking of subunits is largely responsible for the rigidification of Loop1. Furthermore, our pentamer simulations also yield insight into how an incomplete ring could accept the final subunit (Fig. S12 and Movie S3). Altogether, our simulations indicate oligomers smaller than a hexamer (two stacked rings) are not stable due to flexible conformations of Loop1 and Socket1. We note experimental support for this prediction, as our SEC-MALS data shows a stable hexamer in solution (Fig. 3*A*). Thus, our simulations of a TTP pentamer illustrate how the sockets and loops are linked to cooperative formation of intraring and interring interactions.

Finally, we sought to ascertain if Loop1 and Socket1 conformationally control assembly of TTPs from other phages. We chose to examine the TTPs of two siphophages, SPP1 and YSD1, because structures are available (12, 13). Further, this is a good test for the generalization of our findings, as the SPP1 and YSD1 tail tubes consist of true hexameric rings composed of single-domain subunits rather than the trimeric rings of two-domain subunits of P74-26. First, we performed MD simulations of TTP^SPP1^ and TTP^YSD1^ in their monomeric states. Like our TTP^P74-26^ monomer simulations, these simulations predict highly flexible N-terminal and loop residues (Figs. S13 and S14, and Supplementary Movies S4 and S5). In these simulations, the area that contributes to formation of the socket can be blocked by the N-terminal residues and, in the case of YSD1, additionally blocked by the Ig-like domain (Movies S4 and S5). Further, our simulations also predict that the loop (corresponding to Loop1 of P74-26) of TTP^SPP1^ and TTP^YSD1^ folds back into a conformation incompatible with assembly (Figs. S13 and S14).

We next performed MD simulations of TTP^SPP1^ and TTP^YSD1^ in an arrangement of two rings minus a single subunit, similar to our 5-mer simulations of TTP^P74-26^. Like our TTP^P74-26^ pentameric simulation, these simulations suggest that the N-terminal residues (and also the YSD1 Ig-like domain) are rigidified upon assembly, promoting open socket interactions for future interring contacts. Further, the Loop is stabilized by interring and intraring contacts alike. In the case of YSD1, the trend of Loop stabilization follows that of P74-26, where loops stabilized by both interring and intraring contacts are most stable, and only interring contacts provide slightly more stabilization to the loop than only intraring contacts (Fig. S15 and Movie S6). On the other hand, our TTP^SPP1^ 11mer assembly is still highly flexible and preserves little-to-no native contacts between the loop and neighboring subunits (Fig. S16 and Movie S7), suggesting that there may be additional considerations for this assembly. Our simulations of TTP^SPP1^ and TTP^YSD1^ provide supportive evidence that the mechanisms underlying the predicted P74-26 tail tube assembly model are largely conserved across long-tailed phage.

## Discussion

### Mechanisms for cooperative tube assembly

To form tail tube assemblies, individual subunits make extensive interactions within rings and between rings. Within a ring, subunits interact through extended beta sheets and contacts made at Loop1. Between rings, subunits assemble through complementary loop/socket geometry, where the loops of one ring fit snugly into the sockets of an adjacent ring. Notably, this indicates that Loop1 is important for both intraring and interring assembly, consistent with the deletion of this loop completely abrogating assembly *in vitro* (Fig. 4). This highly interwoven network of interactions likely enhances the stability of the entire assembly in a cooperative manner.

How this kind of interlocking network arises from monomer self-assembly is not obvious. As with all phage tails, defects in tail tube assembly would compromise the stability of the tail and ultimately compromise productive phage infection. Because the P74-26 tail tube is exceptionally long, the need to avoid off-pathway assembly is exaggerated, as low-probability defects have more opportunities to arise. Thus, mechanisms that prevent off-pathway assemblies and guide monomers to correct configurations are required, and these mechanisms are likely conserved across many siphophage tail tubes.

Such mechanisms likely regulate each step of self-assembly, including the assembly of monomeric building blocks into trimeric rings, as well as the assembly of rings to higher order structures. We will therefore discuss assembly mechanisms at the level of a monomer, then in the context of intraring interactions, and finally at the level of interring interactions.

### A proposed mechanism for tail tube polymerization

Our high-resolution structure of the P74-26 tail tube reveals specific global- and residue-level interactions important for tube assembly. The most basic building block of the tail tube is a monomer, which our MALS data shows is the predominant species in solution (Fig. 3*A*). Because there is a high population of monomers in solution, we anticipated that the monomer would have mechanisms regulating both intraring and interring contact formation, which is further supported by the faster tail tube formation we observed at higher temperatures. These data indicate that the kinetics of assembly are being controlled by temperature. We hypothesize that the increase in temperature breaks interactions in the monomeric state that hold the monomer in an inactive conformation. The configurational entropy of the assembled tubes is clearly lower than that of the free monomers. As temperature increases, this configurational entropy remains in opposition to assembly. However, at high temperatures, the system also gains a significant amount of conformational entropy within each monomer and can now access assembly-competent conformations. We hypothesize that monomeric TTP prefers an assembly-incompetent state and that the assembly-competent conformation is a higher energy state Therefore, higher thermal energy allows it to more readily populate the assembly-competent state We envision the following nonmutually exclusive mechanisms for regulating this process.

First, our simulations predict that, in the monomeric state, Loop1 can adopt a wide range of meta-stable conformations. Many of these conformations involve Loop1 making contacts with Loop2, precluding both from interacting with other monomers. Because Loop1 is important for both intraring and interring assembly, this mechanism alone can explain the energetic barrier to monomer association; higher temperature can peel Loop1 away from the globular domain and allow it to sample extended states that are competent for binding another subunit’s socket.

**Figure 6:**
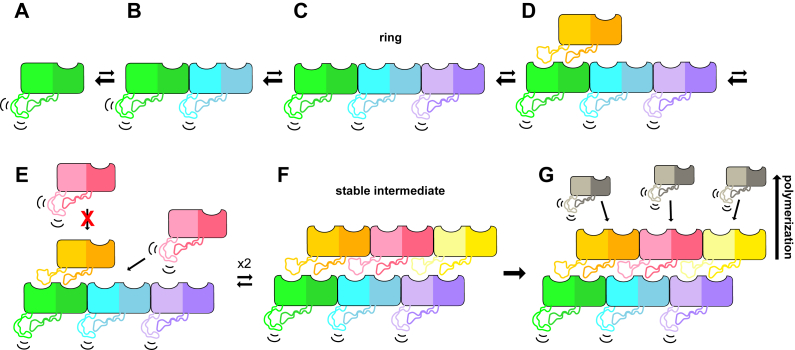
**Proposed model for tail tube assembly.***A*, monomeric TTP in solution has a closed Socket1 and flexible Loop1. *B*, addition of a subunit transiently stabilizes Socket1. *C*, trimeric ring formation leads to opening and stabilization of all Socket1s within the ring, but with flexible Loop1s. *D*, open sockets on the trimer allow for subunit addition. *E*, due to the ball and socket geometry, further subunit addition is likely to occur laterally, completing a ring through the addition of two additional subunits to form (*F*). *F*, a two-ring complex is predicted to be the first stable intermediate, creating a platform for polymerization. *G*, polymerization of the tail tube proceeds spontaneously. TTP, tail tube protein.

Second, we find that intraring interactions control the conformation of Socket1, which thereby controls interring interactions. In our simulations of a TTP monomer, we find that Socket1 is sterically occluded with the N-terminal five residues. However, our simulations of a TTP trimer ring show that Socket1 is unblocked because the N-terminus folds back toward the intraring interface. This reorganization of the N terminus is driven by hydrophobic interactions between the N-terminal methionine and hydrophobic residues that participate in the subunit–subunit interface (Phe123 and Tyr130 in *cis*, and Ala253 and Pro270 in *trans*). Because Met1 plays a critical role in forming the intraring interface, intraring assembly forces the N-terminus to vacate Socket1, which permits Loop1 from another monomer to establish interring contacts. This supports a mechanism by which trimeric rings first form, thereby locking the sockets of all three monomers in a state that promotes interring assembly (Fig. 6*C*). Therefore, the formation of a single ring allows stepwise assembly of the adjacent ring (Fig. 6*D*).

In our *in vitro* assembly system, we do not observe trimers by SEC-MALS, indicating that the trimer is likely not stable. As discussed, our simulations predict that Loop1 is highly flexible even in a trimer, but this loop is only stabilized by the binding of another ring below it. Therefore, the interplay of interring and intraring interactions would mutually stabilize both rings. Thus, a two-ring complex achieves a highly stable scaffold for polymerization to occur from. In fact, we observe a low population of hexamers by SEC-MALS (Fig. 3*A*). Therefore, we predict that two TTP rings is the primary stable intermediate for subsequent assembly in our *in vitro* assembly reactions (Fig. 6*F*). We expect that *in vivo*, the stabilization of a single ring may be achieved by other factors, such as the tail tip complex (TTC) or tail assembly chaperones (TACs). TACs have been shown in phage λ to be essential for phage tail production and to bind both TTP and TMP, thereby creating a scaffold for TTPs on the TMP (30, 31). Thus, in addition to potentially playing a role in TTP stabilization, they are also a likely candidate for the regulation that would need to occur *in vivo* to ensure assembly is occurring in concert with other essential factors like the TMP on a functional timescale.

Once a stable intermediate has formed, tube polymerization is likely to occur by addition of monomers one by one, completing one ring of three subunits through monomer addition before starting to form the next ring (Fig. 6*G*). If the rate-limiting step is the formation of a trimeric ring as suggested by our data, it is unlikely that rings would form in solution and then be added ring-by-ring. Addition of preassembled rings is unlikely as we do not see a buildup of this intermediate in our SEC-MALS data, and our MD simulations suggest this is not a particularly stable intermediate. Rather, monomers are added and thereby stabilized by interring interactions while the intraring interactions form as two more subunits fill in the ring.

In the context of assembly *in vivo*, this mechanism makes sense for multiple reasons. For one, the tail tube must form around the TMP which creates topological constraints that would make ring addition difficult, akin to threading beads onto a string. Our Q167A TTP data further supports our hypothesis of favoring monomer addition as opposed to ring addition, because it is clear that the glutamine residue in WT TTP is serving to prevent the buildup of trimers since they would not be able to get around the TMP. Because a monomer does not have its Socket1 stabilized, it is more likely to bind using its loop face. Thus, our model predicts that tail tubes assemble using a unidirectional polymerization pathway: monomers can only bind to the face of the ring harboring the sockets because the ring’s loops are still flexible. Unidirectional growth could be advantageous in the case of a single polymerization initiation event (at the TTC, for example), from which polymerization extends. Finally, the loop-socket geometry ensures high-fidelity polymerization; it would discourage addition of a monomer to an unfinished ring, preventing aberrant subunit addition and ensuring minimal defects in tube assembly. Based on work done on phage λ, the *in vivo* assembly pathway likely includes the TMP binding the TTC initiator complex, then this complex together with TACs inducing polymerization of TTP around the TMP (31). Once the TTP reaches the end of the TMP, a terminator protein caps the filament to ensure the correct tail length.

The structural conservation between TTPs suggests conserved mechanisms for tube assembly. Our model hinges on the importance of the N-terminus and Loop1 as both autoinhibitory elements in the monomeric state and critical contact points for intraring and interring interactions. Despite the differences in symmetry between P74-26 and other tail tubes, these regions are known to be essential in a number of hexameric systems. In phage λ, a reorganization of the N-terminus and loop occurs between the monomeric and assembled states: the N-terminal region becomes trapped by interactions with the next subunit and the loop is captured along the interring interface (10). These regions are proposed to be critical for tube polymerization in diverse entities including phages T5 and T4, as well as tail-like complexes such as type VI secretion system, pyocins, and extracellular contractile injection systems (5, 8, 9, 10, 12, 32). These observations not only validate our proposed polymerization model but also suggest that these mechanisms are used across a diverse range of phage tail and tail-like assembly pathways.

## Experimental procedures

### Growth and purification of P74-26 virions

P74-26 virions were prepared as previously described (18): P74-26 host strain *T. thermophilus HB8* (ATCC 27634) was grown overnight at 65 °C in *Thermus* growth medium (4 g L^−1^ yeast extract, 8 g L^−1^ tryptone, 3 g L^−1^ NaCl, 1 mM MgCl_2_, and 0.5 mM CaCl_2_). Adsorption was initiated by combining 6 ml of fresh *T. thermophilus* culture with 4 ml of P74-26 phage stock at 1 × 10^6^ plaque-forming units (PFU)/ml and incubated at 65 °C for 10 min. The adsorption reaction was inoculated into 1 L of *Thermus* medium and incubated at 65 °C while shaking for 5 h. Phage particles were dissociated from cell debris with 1 ml chloroform and cell debris was pelleted at 4000*g* for 15 min. The supernatant was incubated with 2 U mL^−1^ DNase I and 1 μg mL^−1^ RNase A for 1 h at 30 °C. To precipitate virions, NaCl was added to a final concentration of 1 M, and PEG 8000 was added to a final concentration of 10% (w/v) while stirring and left to incubate on ice overnight. The next morning, the phage stock was centrifuged at 11, 000*g* for 20 min at 4 °C. Phage pellets were dried, then resuspended in 2 ml of buffer (50 mM Tris pH 7.5, 100 mM NaCl, 1 mM MgSO_4_). 0.4 g solid CsCl was added to each resuspension and the solution was applied to a CsCl step gradient (2 ml steps each of 1.2, 1.3, 1.4, 1.5 g mL^−1^ CsCl and 1 ml cushion of 1.7 g mL^−1^ CsCl, in 50 mM Tris pH 7.5, 100 mM NaCl, 1 mM MgSO_4_). The gradients were spun in a Beckman SW40-Ti rotor at 38,000 RPM for 18 h at 4 °C. The virion layer was isolated from the gradient and dialyzed twice into 2 L of 50 mM Tris pH 8.0, 10 mM NaCl, 10 mM MgCl_2_ at 4 °C. P74-26 virions were then concentrated to 1 × 10^12^ PFU mL^−1^.

### Cloning and mutagenesis of P74-26 TTP

The P74-26 TTP (gp93) gene was *E. col*i–codon-optimized and synthesized by GenScript, then subcloned into a pSMT3 vector with a cleavable N-terminal 6× His-SUMO tag. Restriction enzymes were purchased from New England BioLabs, and oligonucleotide primers were purchased from Integrated DNA Technologies (Table S1). Mutations were introduced using the QuikChange protocol.

### Expression and purification of P74-26 TTP

ArcticExpress (DE3) cells (Agilent) were transformed with TTP-pSMT3 and grown at 37 °C on a kanamycin (30 μg/ml) plate. A single colony was used to inoculate a 50 ml culture of 2xYT media (per liter: 16 g Bacto tryptone, 10 g yeast extract, 5 g NaCl) which was grown overnight at 37 °C in the presence of kanamycin (30 μg/ml) and gentamicin (20 μg/ml) to select for the desired plasmid and Cpn10/Cpn60 chaperonins, respectively. The overnight culture was then used to inoculate 1 L cultures which were grown at 30 °C without antibiotics for 3 h while shaking, allowed to cool at 4 °C for 10 min, induced with a final concentration of 1 mM IPTG, and then incubated at 12 °C overnight with shaking. Such temperatures were used in accordance with the cell type, as well as to reduce toxic tube formation leading to a decrease in the yield of soluble protein. Pelleted bacteria were then resuspended in buffer A (50 mM Tris pH 8.0, 300 mM KCl, 20 mM imidazole, 5 mM βME, 10% glycerol) and lysed by high pressure cell disruption on ice. The lysate was centrifuged at 23,000*g* for 40 min at 4 °C. All subsequent steps were performed on ice in a cold room. The supernatant was filtered through a 0.45-μm filter (Millex, EMD Millipore) and loaded onto a 5 ml Hi-trap Ni-nitrilotriacetic acid column (Cytiva) that was preequilibrated in buffer A. Following lysate loading, the column was washed with 5 column volumes of buffer A, then the protein was eluted in buffer B (50 mM Tris pH 8.0, 300 mM KCl, 0.5 M imidazole, 5 mM βME, 10% glycerol). Two hundred fifty microliters of ULP1 protease were added to the eluate to cleave the tag, then dialyzed 1:1000 against buffer A overnight. The protein was then subjected to a subtractive step over buffer A–equilibrated nickel columns to separate TTP from the tags. The protein was concentrated by centrifugal filtration (Amicon, EMD Millipore, 10-kDa MW cutoff) at 4 °C. Final protein concentration was determined by UV absorbance at 280 nm, using an extinction coefficient of 23,380 M^−1^ cm^−1^.

### Electron microscopy

#### Negative stain EM

Carbon-coated 200 mesh copper grids (Electron Microscopy Sciences) were glow discharged on a PELCO easiGlow (Ted Pella) at 20 mA for 60 s (negative polarity). 3.5 μl of sample was applied to the grid and incubated for 1 min. Excess sample was blotted, then the grid was washed with water followed by staining with 1% uranyl acetate (pH 4.5). Grids were viewed with a Philips CM120 electron microscope at 120 kV on a Gatan Orius SC1000 camera. Micrographs were collected between 400,00× and 660,00×.

#### Cryo-EM sample preparation

Grids were glow discharged on a PELCO easiGlow (Ted Pella) at 25 mA for 60 s (negative polarity). *Virion tails*: 3.5 μl of purified virions at 1 × 10^10^ PFU mL^−1^ was applied to a 400-mesh C-flat holey carbon-coated grid (Electron Microscopy Sciences) at 10 °C with 90% humidity in a Vitrobot Mark IV (FEI). Sample was blotted from both sides for 10 s after a wait time of 15 s, then immediately vitrified by plunging into liquid ethane. In vitro–assembled tubes: 3.5 μl of purified TTP at 1.5 mg mL^-1^ was applied to a 400-mesh copper lacey carbon-coated grid (Electron Microscopy Sciences) at 10 °C with 90% humidity in a Vitrobot Mark IV (FEI). Sample was manually blotted and another 3.5 μl of sample was applied to the grid. Sample was then blotted from both sides for 8 s after a wait time of 15 s, then immediately vitrified by plunging into liquid ethane.

#### Cryo-EM data collection

Micrographs were collected on a 200 kV Talos-Arctica electron microscope (FEI) equipped with a K3 Smit direct electron detector (Gatan). *Virion tails*: Images were collected at a magnification of 45,000 in super-resolution mode with an unbinned pixel size of 0.435 Å per pixel and a total dose of 37.9644 e^−^ Å^−2^ per micrograph, with a target defocus range of −0.5 to −1.6 μm. In total, 2127 micrographs were collected. *In vitro–assembled tubes:* Images were collected as above with a total dose of 38.4312 e^−^ Å^−2^ per micrograph. In total, 2030 micrographs were collected.

#### Data processing

Micrograph frames were aligned in IMOD with 2× binning, resulting in a pixel size of 0.87 Å per pixel. Initial contrast transfer function estimation was done using CTFFind4 within CryoSPARC. All the following steps were done within CryoSPARC (19). *Virion tail dataset*: 217 particles were manually picked for a training dataset. From these particles, five classes were formed and two were selected as templates for the filament tracer. Filament tracer was performed with a 100 Å filament diameter and 0.4 diameters between segments. A total of 795,534 particles were extracted with a box size of 300 pixels and classified into 100 classes. A total of 787,734 particles in 52 classes were used for an *ab initio* helical refinement with C1 symmetry. The resulting volume underwent consecutive rounds of symmetry searches followed by homogenous refinements until the helical parameters were determined with local search minima of a −44° twist and a 40 Å rise. These parameters were used along with enforced C3 symmetry in the helical refinement. Local CTF refinement was performed on the 787,734 input particles and the map from the previous job was used for a final helical refinement. The Guinier plot from this job was used to obtain a B-factor value of −182.3 to sharpen the final map for a resolution of 2.73 Å. *In vitro–assembled tubes dataset*: 221 particles were manually picked for a training dataset. From these particles, five classes were formed and three were selected as templates for the filament tracer. Filament tracer was performed with a 100 Å filament diameter and 0.4 diameters between segments. A total of 619,907 particles were extracted with a box size of 300 pixels and classified into 100 classes. A total of 395,397 particles in 74 classes were used for a helical refinement using the helical refinement and symmetry determined above. Local CTF refinement was performed and the map was sharpened with a B-factor of −136.8 for a final resolution of 2.81 Å.

### Model building and refinement

Gp93 was *de novo* built into a single subunit of the cryo-EM density of the virion tail map in Coot (33). Sidechain density was obvious due to the high resolution, so easily identifiable aromatic residues and the known sequence of gp93 were used to obtain residue register. For the model of *in vitro*–assembled tubes, the gp93 model was fitted into the *in vitro*–assembled tube map density and then manually refined. Both models were then refined into their respective maps using the Phenix real-space refine procedure (34). The Isolde plugin (35) in ChimeraX (36) was then used to further refine the map into the model followed by a final Phenix real-space refinement. The real-space refinement statistics are listed in Table S2.

### Size exclusion chromatography-multi angle light scattering

TTP was run on a tandem SEC-MALS detector by injecting 100 μl sample at a concentration of 1 mg/ml using a 1260 Infinity HPLC (Agilent). The column was preequilibrated in 0.1-μm filtered buffer containing 50 mM Tris pH 8.0, 150 mM KCl, 5 mM βME, 5% glycerol, and the sample was filtered through a 0.22-μm spin filter before loading. Sample was on ice until directly prior to loading, then the run itself was done at room temperature. Elution was monitored by a Dawn Heleos-II MALS detector and an Optilab T-rex differential refractive index detector (Wyatt Technology). Peaks were defined and analyzed in ASTRA6 (Wyatt Technology).

### Light scattering of tube assembly

Kinetics of tube assembly for WT and mutant TTP constructs were observed by monitoring changes in light scattering using a FluoroMax-4 spectrofluorometer (Horiba Scientific) at a wavelength of 350 nm with a 1-nm excitation bandpass and a 0.5-nm emission bandpass. Samples were loaded into a 75 μl quartz fluorometer cuvette (Starna Cells 16.50F-Q-10/Z15) with 125 μl to bring the sample meniscus above the window aperture. For each experiment, the fluorometer was programmed to equilibrate at the target temperature for 120 s with the cuvette in the chamber, samples were loaded from ice into the cuvette, and the program was initiated to take reads every 30 s with shutters closing between reads. The sample was filtered through a 0.22-μm filter directly prior to initiating the experiment to filter out any spontaneous tube formation. All experiments were performed at ∼1 mg/ml unless otherwise noted.

Raw light scattering data was scaled to zero by subtracting the first value from all consecutive values so that the amplitudes of the curves could be compared directly. Light scattering curves were fit depending on the shape of the curve using GraphPad Prism version 7.04 (https://www.graphpad.com/scientific-software/prism/). Linear slopes were fit linearly, while nonlinear slopes were fit with either a one phase association (Y=Y0 + (Plateau-Y0)∗(1-exp(-K∗x))) or a two phase exponential association equation (Y= amplitude1∗(1-exp(-K1∗X)) + amplitude2∗(1-exp(-K2∗X))). Curves with a sigmoidal shape were fit to (Y=Bottom + (Top-Bottom)/(1+10ˆ((LogIC50-X)∗Slope))) to account for their sigmoidal shape. The second derivative of this fit was taken in order to determine the point of inflection for sigmoidal curves, and that point was used as the location where a linear slope could be fit to the fast rate (Fig. S7).

### Electrostatic calculations

Surface electrostatics were calculated in PyMol using the Adaptive Poisson-Boltzmann Solve plugin (https://pymolwiki.org/index.php/APBS_Electrostatics_Plugin) (37).

### MD simulations

#### Simulation preparation

Initial structures and topology files were generated with the *tleap* module of AMBERTOOLS20 (38). All initial coordinate and topology files are included in the supplementary information. Protein interactions were described with the Amber ff19SB force field (39). Proteins were centered in a truncated octahedral periodic box solvated with the optimal point charge four-point water model with minimum 14 Å padding (40). Sodium ions were added to make each system charge-neutral, and additional salt was added to reach 0.15 M concentration. Hydrogen mass repartitioning was applied with ParMed (41), increasing the weight of solute hydrogen atoms to 3.024 Da. P74-26 TTP starting structures were taken from those described herein, YSD1 TTP were taken from PDB 6XGR, and SPP1 TTP were taken from PDB 6YEG (12, 13). In simulations of SPP1 TTP, C-terminal residues were found to be very flexible in the absence of interring interactions. Thus, for simulations of rings, we truncated residues 153-176 of the “top” ring to avoid their interaction with subunits across the periodic boundary. Further, we used additional padding (up to 22 Å) for this system. To ensure our predictions were not force field dependent, we also conducted a P74-26 TTP monomer simulation with the CHARMM36m protein force field and CHARMM-modified TIP3P water model (42). This system was initialized using the CHARMM-GUI webserver (43). For this system, the protein was centered in a rectangular periodic box with 14 Å padding, made charge-neutral, and additional salt added up to 0.15 M. Hydrogen mass repartitioning was also applied to this system.

#### Simulation methodology

All simulations were performed in the gpu-accelerated *pmemd* module of the AMBER20 simulation package (44). All input files are included in the supplement. Systems were energy minimized for 500 steps of steepest descent and conjugate gradient. Systems were heated from 100 K to 310 K over 500 ps in the canonical (NVT) ensemble. Independent simulations were seeded by drawing different random initial velocities from the Maxwell-Boltzmann distribution. During heating, the temperature was controlled with the Langevin thermostat with a 1.0 ps^-1^ friction coefficient (45). A 9 Å explicit cutoff was applied, and the Particle-Mesh Ewald method was used to correct long range interactions. For the CHARMM36m monomer simulation, electrostatic interactions were calculated with a force-switching scheme, with a switching distance of 10 A and explicit cutoff of 12 A. With hydrogen mass repartitioning applied, we used a 4-fs integration timestep to propagate the equations of motion. Bonds connecting hydrogen atoms to heavy atoms were constrained with the SHAKE algorithm (46). After heating, systems were simulated in the isothermal-isobaric (NPT) ensemble at 310 K and 1 bar using the Langevin thermostat and Monte Carlo barostat (47). Simulations were run in triplicate for varying times depending on system size, with at least ∼1 to 2 μs each for P74-26 TTP systems (Table S5).

#### Simulation analysis

K-means clustering (*k* = 10) was performed with the cpptraj program on each simulation (48). Representative frames from the largest cluster are included as PDB files in the supplement. Distances and RMS fluctuations (RMSF) were calculated using cpptraj with the *distance* and *atomicfluct* commands, respectively. Distances were calculated using centers of mass to avoid fluctuations that arise from internal conformational changes. RMSF was calculated by first aligning the trajectories about the β-sandwich domain of subunits (hence, loops were not included in the alignment) and then calculating a per-residue RMSF by averaging the backbone C, Cα, O, and N values. Native contacts were calculated using the *nativecontacts* command in cpptraj; the initial cryo-EM reconstructions were used as the reference structure that defined native contacts. Native contacts were calculated including only the heavy atoms in Loop1 and all the heavy atoms in the neighboring subunit(s). Supplemental movies were generated with VMD 1.9.4a55 (https://www.ks.uiuc.edu/Development/Download/download.cgi?PackageName=VMD) (49).

## Data availability

Cryo-EM maps and corresponding models have been deposited to the Electron Microscopy Data Bank (accession codes EMD-28026 and EMD-28042) and PDB (accession codes 8ED0 and 8EDX). The plasmid for protein production of P74-26 TTP (gp93) has been deposited to Addgene (https://www.addgene.org/browse/article/28229425/). Representative molecular dynamic simulations have been included as supplemental files. Full trajectories available upon request.

## Supporting information

This article is accompanied by supporting information (9, 12, 13, 14, 15, 18, 23, 50, 51, 52, 53, 54, 55, 56, 57, 58, 59, 60, 61, 62)

## Conflict of interest

The authors declare that they have no conflicts of interest with the contents of this article.

## Supporting information

Supporting Information

2rings_viriontails

2rings_vitrotubes

heat_hmr_310.in

md_hmr.in

min.in

p74-26_5mer.pdb

p74-26_5mer_hmr.inpcrd

p74-26_5mer_hmr.prmtop

P74-26_5mer_trajectory_labeled

p74-26_monomer.pdb

p74-26_monomer_c36m_hmr.parm7

p74-26_monomer_c36m_hmr.rst7

p74-26_monomer_ff19sb_hmr.inpcrd

p74-26_monomer_ff19sb_hmr.prmtop

p74-26_monomer_trajectory_labeled

p74-26_trimer.pdb

p74-26_trimer_hmr.inpcrd

p74-26_trimer_hmr.prmtop

P74-26_trimer_trajectory_labeled

spp1_11mer.pdb

spp1_11mer_hmr.inpcrd

spp1_11mer_hmr.prmtop

spp1_11mer_trajectory_labeled

spp1_monomer.pdb

spp1_monomer_hmr.inpcrd

spp1_monomer_hmr.prmtop

spp1_monomer_trajectory_labeled

ysd1_11mer.pdb

ysd1_11mer_hmr.inpcrd

ysd1_11mer_hmr.prmtop

ysd1_11mer_trajectory_labeled

ysd1_monomer.pdb

ysd1_monomer_hmr.inpcrd

ysd1_monomer_hmr.prmtop

ysd1_monomer_trajectory_labeled

## References

[1] Fokine A., Rossmann M.G. (2014). Molecular architecture of tailed double-stranded DNA phages. Bacteriophage.

[2] Linares R., Arnaud C.A., Degroux S., Schoehn G., Breyton C. (2020). Structure, function and assembly of the long, flexible tail of siphophages. Curr. Opin. Virol..

[3] Davidson A.R., Cardarelli L., Pell L.G., Radford D.R., Maxwell K.L. (2012). Long noncontractile tail machines of bacteriophages. Adv. Exp. Med. Biol..

[4] Zinke M., Schröder G.F., Lange A. (2022). Major tail proteins of bacteriophages of the order Caudovirales. J. Biol. Chem..

[5] Pell L.G., Kanelis V., Donaldson L.W., Howell P.L., Davidson A.R. (2009). The phage λ major tail protein structure reveals a common evolution for long-tailed phages and the type VI bacterial secretion system. Proc. Natl. Acad. Sci. U. S. A..

[6] Veesler D., Cambillau C. (2011). A common evolutionary origin for tailed-bacteriophage functional modules and bacterial machineries. Microbiol. Mol. Biol. Rev..

[7] Plisson C., White H.E., Auzat I., Zafarani A., São-José C., Lhuillier S. (2007). Structure of bacteriophage SPP1 tail reveals trigger for DNA ejection. EMBO J..

[8] Langlois C., Ramboarina S., Cukkemane A., Auzat I., Chagot B., Gilquin B. (2015). Bacteriophage SPP1 tail tube protein self-assembles into β-structure-rich tubes. J. Biol. Chem..

[9] Arnaud C.A., Effantin G., Vivès C., Engilberge S., Bacia M., Boulanger P. (2017). Bacteriophage T5 tail tube structure suggests a trigger mechanism for Siphoviridae DNA ejection. Nat. Commun..

[10] Campbell P.L., Duda R.L., Nassur J., Conway J.F., Huet A. (2020). Mobile loops and electrostatic interactions maintain the flexible tail tube of bacteriophage lambda. J. Mol. Biol..

[11] Zinke M., Fricke P., Samson C., Hwang S., Wall J.S., Lange S. (2017). Bacteriophage tail-tube assembly studied by proton-detected 4D solid-state NMR. Angew. Chem. Int. Ed..

[12] Zinke M., Sachowsky K.A.A., Öster C., Zinn-Justin S., Ravelli R., Schröder G.F. (2020). Architecture of the flexible tail tube of bacteriophage SPP1. Nat. Commun..

[13] Hardy J.M., Dunstan R.A., Grinter R., Belousoff M.J., Wang J., Pickard D. (2020). The architecture and stabilisation of flagellotropic tailed bacteriophages. Nat. Commun..

[14] Bárdy P., Füzik T., Hrebík D., Pantůček R., Thomas Beatty J., Plevka P. (2020). Structure and mechanism of DNA delivery of a gene transfer agent. Nat. Commun..

[15] Kizziah J.L., Manning K.A., Dearborn A.D., Dokland T. (2020). Structure of the host cell recognition and penetration machinery of a Staphylococcus aureus bacteriophage. PLoS Pathog..

[16] Yu M.X., Slater M.R., Ackermann H.W. (2006). Isolation and characterization of Thermus bacteriophages. Arch. Virol..

[17] Minakhin L., Goel M., Berdygulova Z., Ramanculov E., Florens L., Glazko G. (2008). Genome comparison and proteomic characterization of Thermus thermophilus bacteriophages P23-45 and P74-26: siphoviruses with triplex-forming sequences and the longest known tails. J. Mol. Biol..

[18] Stone N.P., Demo G., Agnello E., Kelch B.A. (2019). Principles for enhancing virus capsid capacity and stability from a thermophilic virus capsid structure. Nat. Commun..

[19] Punjani A., Rubinstein J.L., Fleet D.J., Brubaker M.A. (2017). cryoSPARC: algorithms for rapid unsupervised cryo-EM structure determination. Nat. Met..

[20] Papadopoulos S., Smith P.R. (1982). The structure of the tail of the bacteriophage φCbK. J. Ultrasruct. Res..

[21] Leonard K.R., Kleinschmidt A.K., Lake J.A. (1973). Caulobacter crescentus bacteriophage phiCbK: structure and *in vitro* self-assembly of the tail. J. Mol. Biol..

[22] González B., Li D., Li K., Wright E.T., Hardies S.C., Thomas J.A. (2021). Structural studies of the phage G tail demonstrate an atypical tail contraction. Viruses.

[23] Hardy J.M., Dunstan R.A., Lithgow T., Coulibaly F. (2022). Tall tails: cryo-electron microscopy of phage tail DNA ejection conduits. Biochem. Soc. Trans..

[24] Sassi M., Bebeacua C., Drancourt M., Cambillau C. (2013). The first structure of a Mycobacteriophage, the Mycobacterium abscessus subsp. bolletii phage Araucaria. J. Virol..

[25] Kong X.P., Onrust R., O’Donnell M., Kuriyan J. (1992). Three-dimensional structure of the beta subunit of E. coli DNA polymerase III holoenzyme: a sliding DNA clamp. Cell.

[26] Krishna T.S., Kong X.P., Gary S., Burgers P.M., Kuriyan J. (1994). Crystal structure of the eukaryotic DNA polymerase processivity factor PCNA. Cell.

[27] Kuriyan J., O’Donnell M. (1993). Sliding clamps of DNA polymerases. J. Mol. Biol..

[28] Mahony J., Alqarni M., Stockdale S., Spinelli S., Feyereisen M., Cambillau C. (2016). Functional and structural dissection of the tape measure protein of lactococcal phage TP901-1. Sci. Rep..

[29] Katsura I., Tsugita A. (1977). Purification and characterization of the major protein and the terminator protein of the bacteriophage lambda tail. Virology.

[30] Maxwell K.L., Davidson A.R. (2014). A shifty chaperone for phage tail assembly. J. Mol. Biol..

[31] Xu J., Hendrix R.W., Duda R.L. (2014). Chaperone–protein interactions that mediate assembly of the bacteriophage lambda tail to the correct length. J. Mol. Biol..

[32] Lim Y.T., Jobichen C., Wong J., Limmathurotsakul D., Li S., Chen Y. (2015). Extended loop region of Hcp1 is critical for the assembly and function of type VI secretion system in Burkholderia pseudomallei. Sci. Rep..

[33] Emsley P., Cowtan K. (2004). Coot: model-building tools for molecular graphics. Acta Crystallogr. D Biol. Crystallogr..

[34] Adams P.D., Afonine P.V., Bunkóczi G., Chen V.B., Davis I.W., Echols N. (2010). Phenix: a comprehensive Python-based system for macromolecular structure solution. Acta Crystallogr. D Biol. Crystallogr..

[35] Croll T.I. (2018). Isolde: a physically realistic environment for model building into low-resolution electron-density maps. Acta Crystallogr. D Struct. Biol..

[36] Pettersen E.F., Goddard T.D., Huang C.C., Meng E.C., Couch G.S., Croll T.I. (2021). UCSF ChimeraX: structure visualization for researchers, educators, and developers. Protein Sci..

[37] Baker N.A., Sept D., Joseph S., Holst M.J., McCammon J.A. (2001). Electrostatics of nanosystems: Application to microtubules and the ribosome. Proc. Natl. Acad. Sci. U. S. A..

[38] D A., Metin Aktulga H., Belfon K., Ben-Shalom I., Brozell S.R., Cerutti D.S. (2020).

[39] Tian C., Kasavajhala K., Belfon K.A.A., Raguette L., Huang H., Migues A.N. (2020). ff19SB: amino-Acid-Specific protein backbone parameters trained against quantum mechanics energy surfaces in solution. J. Chem. Theor. Comput..

[40] Izadi S., Anandakrishnan R., Onufriev A.V. (2014). Building water models: a different approach. J. Phys. Chem. Lett..

[41] Shirts M.R., Klein C., Swails J.M., Yin J., Gilson M.K., Mobley D.L. (2017). Lessons learned from comparing molecular dynamics engines on the SAMPL5 dataset. J. Comput. Aided Mol. Des..

[42] Huang J., Rauscher S., Nawrocki G., Ran T., Feig M., de Groot B.L. (2017). CHARMM36m: an improved force field for folded and intrinsically disordered proteins. Nat. Met..

[43] Jo S., Kim T., Iyer V.G., Im W. (2008). CHARMM-GUI: a web-based graphical user interface for CHARMM. J. Comput. Chem..

[44] Salomon-Ferrer R., Götz A.W., Poole D., Le Grand S., Walker R.C. (2013). Routine microsecond molecular dynamics simulations with AMBER on GPUs. 2. Explicit solvent particle mesh Ewald. J. Chem. Theor. Comput..

[45] Loncharich R.J., Brooks B.R., Pastor R.W. (1992). Langevin dynamics of peptides: the frictional dependence of isomerization rates of *N*-acetylalanyl-*N*′-methylamide. Biopolymers.

[46] Ryckaert J.P., Ciccotti G., Berendsen H.J.C. (1977). Numerical integration of the cartesian equations of motion of a system with constraints: molecular dynamics of n-alkanes. J. Comput. Phys..

[47] Åqvist J., Wennerström P., Nervall M., Bjelic S., Brandsdal B.O. (2004). Molecular dynamics simulations of water and biomolecules with a Monte Carlo constant pressure algorithm. Chem. Phys. Lett..

[48] Roe D.R., Cheatham T.E. (2013). PTRAJ and CPPTRAJ: software for processing and analysis of molecular dynamics trajectory data. J. Chem. Theor. Comput..

[49] Humphrey W., Dalke A., Schulten K. (1996). Vmd: visual molecular dynamics. J. Mol. Graph.

[50] Inamdar M.M., Gelbart W.M., Phillips R. (2006). Dynamics of DNA ejection from bacteriophage. Biophys. J..

[51] Zheng W. (2017). Refined Cryo-EM structure of the T4 tail tube: exploring the lowest dose limit. Structure.

[52] Tigerström A. (2005). The Biologist’s Forum: Thermostability of proteins. BIOS.

[53] Scandurra R., Consalvi V., Chiaraluce R., Politi L., Engel P.C. (1998). Protein thermostability in extremophiles. Biochimie.

[54] Gromiha M.M., Pathak M.C., Saraboji K., Ortlund E.A., Gaucher E.A. (2013). Hydrophobic environment is a key factor for the stability of thermophilic proteins. Proteins.

[55] Szilágyi A., Závodszky P. (2000). Structural differences between mesophilic, moderately thermophilic and extremely thermophilic protein subunits: results of a comprehensive survey. Structure.

[56] Zhou H.-X., Dong F. (2003). Electrostatic contributions to the stability of a thermophilic cold shock protein. Biophys. J..

[57] Vogt G., Woell S., Argos P. (1997). Protein thermal stability, hydrogen bonds, and ion pairs. J. Mol. Biol..

[58] Pietilä M.K. (2013). Insights into head-tailed viruses infecting extremely halophilic archaea. J. Virol..

[59] Ptchelkine D. (2017). Unique architecture of thermophilic archaeal virus APBV1 and its genome packaging. Nat. Commun..

[60] Dimaio F. (2015). A virus that infects a hyperthermophile encapsidates A-form DNA. Science.

[61] Mary H., Brouhard G.J. (2019). Kappa (κ): Analysis of curvature in biological image data using B-splines. bioRxiv.

[62] Krissinel E., Henrick K. (2007). Protein interfaces, surfaces and assemblies’ service PISA at the European Bioinformatics Institute. Inference of macromolecular assemblies from crystalline state. J. Mol. Biol..

